# Association of Apolipoprotein C-III Gene Polymorphisms (rs2854116 and rs2854117) with Susceptibility to Metabolic Dysfunction-Associated Steatotic Liver Disease (MASLD) in a Turkish Population

**DOI:** 10.3390/medicina61081479

**Published:** 2025-08-18

**Authors:** Damla Karaagac, Suat Morkuzu, Naci Senkal, Ersel Bilgin, Yasemin Oyacı, Tufan Tükek, Sacide Pehlivan, Alpay Medetalibeyoglu

**Affiliations:** 1 Department of Internal Medicine, Istanbul Medical Faculty, Istanbul University, 34452 İstanbul, Turkey; damlaaltunok26@gmail.com (D.K.);; 2 Department of Family Medicine, Istanbul Medical Faculty, Istanbul University, 34452 İstanbul, Turkey; 3 Department of Gastroenterology and Hepatology, Istanbul Medical Faculty, Istanbul University, 34452 İstanbul, Turkey; 4 Department of Medical Biology, Istanbul Medical Faculty, Istanbul University, 34452 İstanbul, Turkey

**Keywords:** MASLD, non-alcoholic fatty liver disease, metabolic syndrome, apolipoprotein C-III, NAFLD

## Abstract

*Background and Objectives*: Metabolic dysfunction-associated steatotic liver disease (MASLD) is characterized by the accumulation of fat in the liver, progressing from simple steatosis to various complications, with increasing prevalence in the modern world. Our study aimed to investigate the relationship between MASLD pathogenesis and the presence of apolipoprotein C-III (ApoC-III) gene variants rs2854116 and rs2854117 by comparing allele and genotype frequencies between MASLD patients and healthy individuals, as well as analyzing their association with biochemical parameters in Turkish populations. *Materials and Methods*: The study included 202 MASLD patients and 100 healthy controls who presented to our outpatient clinic. MASLD presence was determined by ultrasonography (USG). The demographic, laboratory, and clinical data of the participants were recorded. ApoC-III gene variants rs2854116 and rs2854117 were genotyped using the Polymerase Chain Reaction-Restriction Fragment Length Polymorphism (PCR-RFLP) method from genomic DNA samples obtained from blood. *Results*: The genotype and allele frequencies of ApoC-III gene variants rs2854116 and rs2854117 did not show significant differences between patient and healthy groups (*p* > 0.05). When biochemical parameters were evaluated, the LDH value of rs2854116 variant CT/CC genotype carriers was found to be significantly higher than TT genotype carriers (*p* = 0.016). *Conclusions*: We observed a high prevalence of MASLD in our Turkish cohort. However, the specific genetic variants we investigated were not associated with MASLD status. This suggests that these variants may not be significant contributing factors to MASLD in this population.

## 1. Introduction

Metabolic dysfunction-associated steatotic liver disease (MASLD) is the current term for what was formerly called nonalcoholic fatty liver disease (NAFLD). MASLD refers to a condition characterized by hepatic steatosis involving ≥5% of hepatocytes in the absence of a defined secondary cause of fat accumulation, such as medications or monogenic disorders [1].

The global prevalence of MASLD has been estimated at 30.2–32.4% [2,3]. The pathophysiological spectrum of MASLD progresses from non-alcoholic fatty liver (NAFL), characterized by macro vesicular steatosis with minimal inflammation, to non-alcoholic steatohepatitis (NASH), marked by inflammatory infiltrates and hepatocellular ballooning, and ultimately to cirrhosis, defined by fibrous septation and nodular regeneration. These stages represent a continuum of increasing hepatocellular injury and functional impairment, ultimately contributing to significant morbidity and mortality [4,5].

In a study conducted in Turkey, the prevalence of MASLD was found to be 48.3% [6]. MASLD is commonly associated with a range of comorbid conditions, most notably obesity, type 2 diabetes mellitus, hypertension, dyslipidemia, atherosclerotic cardiovascular disease, and heart failure [4,7]. Given these associations, identifying risk factors is important for stratifying patients and understanding the mechanisms of disease progression, which can inform clinical management.

Apolipoproteins are associated with lipoproteins, which play a crucial role in the transport of cholesterol, triglycerides, phospholipids, and fat-soluble vitamins between the intestine, liver, and peripheral tissues. Apo C-III is a low molecular weight apolipoprotein primarily produced by hepatic cells and, to a lesser extent, by enterocytes. Plasma levels of ApoC-III are elevated in patients with diabetes mellitus (DM) and are associated with insulin resistance [8,9]. This suggests that the increase in ApoC-III during the development of atherogenic dyslipidemia may exacerbate insulin resistance, thereby accelerating the development of type 2 DM and hepatic steatosis [10,11,12]. Recent studies on ApoC-III have highlighted that two single nucleotide polymorphisms (SNPs) in the gene encoding ApoC-III (rs2854116 and rs2854117) may be associated with hypertriglyceridemia [13,14,15,16,17]. These SNPs are in the promoter region of the gene. The variant alleles of rs2854116 (T-455C) and rs2854117 (C-482T) are linked to higher in vitro expression of ApoC-III compared to the wild-type alleles [18]. These ApoC-III variants have been shown to promote both fasting and postprandial hypertriglyceridemia by reducing triglyceride clearance. Specifically, the ApoC-III variants (rs2854116 and rs2854117) result in an approximately 30% increase in fasting plasma ApoC-III concentrations, a 60% increase in fasting plasma triglyceride concentrations, and nearly a twofold increase in postprandial plasma triglyceride and retinyl fatty acid ester concentrations following an oral fat tolerance test [8].

In our study, we aim to investigate the association of the ApoC-III gene variants rs2854116 and rs2854117 with the presence and pathogenesis of MASLD in the Turkish population. This will be accomplished by comparing the allele and genotype frequencies of these variants in individuals with MASLD to those of healthy controls. Additionally, we will analyze the biochemical parameters of the participants. This research will contribute to population-specific genetic data.

## 2. Materials and Methods

### 2.1. Patients and Laboratory Findings

Volunteers over 18 years of age were included in our study, comprising patients diagnosed with MASLD through imaging methods and healthy controls without any known disease history, who presented as outpatients to the Internal Medicine Clinic of Istanbul University Istanbul Faculty of Medicine Hospital between May–September 2023. Individuals over 75 years of age; under 18 years of age; those with active malignancy or history thereof; those with any organ-tissue transplant history; pregnant women; those consuming alcohol over 140 g weekly for women and 210 g for men; those with glomerular filtration rate (GFR) < 60; pregnant women; those with CRP > 5 mg/L; those with primary liver diseases other than MASLD such as primary biliary cholangitis, autoimmune hepatitis, viral hepatitis, and Wilson’s disease; those with active infection; and those with missing information were excluded from the study. When volunteers were included in the study, their current liver steatosis status was re-evaluated using liver ultrasonography, and patients without current steatosis and controls with steatosis were excluded from the study. After exclusions, 202 confirmed MASLD patients and 100 healthy controls were included in the study.

This study protocol was approved by the Non-Scope Clinical Research Ethics Committee of Istanbul University Istanbul Faculty of Medicine (Date: 14 April 2023) and was conducted in accordance with the standards specified in the World Medical Association Declaration of Helsinki. Informed consent forms were obtained from all patients and healthy volunteers before starting the study.

Participants’ weekly alcohol consumption, smoking habits, hypertension (HT), diabetes mellitus (DM), DM type, DM complications, impaired fasting glucose, impaired glucose tolerance, dyslipidemia and its types, cardiovascular disease (CVD), cerebrovascular disease, family history of CVD, medication groups for DM and HT, and disease durations were questioned and recorded. Laboratory tests performed from blood samples given by participants after 12 h of fasting during their outpatient clinic control and recorded in the outpatient file were evaluated. Those with triglyceride (TG) levels ≥ 150 mg/dL were defined as having hypertriglyceridemia (hyper-TG). Those who met any of the definitions of hyper-TG, hypo-HDL, and hyper-LDL were combined under the definition of dyslipidemia. FIB-4 [19], NFS [20], homeostatic model assessment for insulin resistance (HOMA-IR) [21], and De Ritis ratio (AST/ALT) [22] were calculated using laboratory values, clinical characteristics, anthropometric measurements, and demographic features of the patients.

### 2.2. Determination of Liver Steatosis Grade

After completing the anthropometric measurements of individuals, a high-resolution ultrasonography system B mode (GE Logiq E9, Milwaukee, WI, USA) and a convex probe with a frequency bandwidth of 1–6 MegaHertz were used to evaluate the presence of liver steatosis and determine the degree of steatosis if present. The grading of liver steatosis is generally obtained using certain USG features including liver brightness, contrast difference between liver and kidney, appearance of intrahepatic vessels on USG, and liver parenchyma and diaphragm. Steatosis grades were determined as grade 0 normal liver, grade 1 mild steatosis, grade 2 moderate steatosis, and grade 3 severe steatosis. The performance of USG in detecting mild steatosis (fat content > 5%) is low, with a sensitivity of 60.9–65% [23,24]. When compared with biopsy for the detection of moderate and severe liver steatosis (>20–30% fatty), sensitivity was found to be 84.8% and specificity 93.6% [25]. In our study, the definition of steatosis used when defining MASLD was based on steatosis detected in USG.

### 2.3. APOC-III Gene rs2854116 and rs2854117 Variant Analyses

From blood samples taken from the study groups in 2 tubes containing 3 cc of Ethylenediaminetetraacetic acid (EDTA), leukocyte isolation was performed first, then DNA isolation was carried out from the obtained leukocytes using a commercial kit (Elk Biotech Cat no: EP008) (ELK Biotecnology Co., Ltd., Denver, CO, USA). ApoC-III rs2854116 and rs2854117 variants were genotyped by the Polymerase Chain Reaction-Restriction Fragment Length Polymorphism (PCR-RFLP) method. FokI enzyme was used as the restriction endonuclease enzyme for the rs2854116 variant, and MspI enzyme was used for the rs2854117 variant (New England Biolabs, cat no: R0109S, R0106T, respectively) (New England Biolabs, Ipswich, MA, USA). Restriction products were run on 3.5% agarose gel and genotyping was performed under UV light.

### 2.4. Statistical Analysis

Statistical analysis of the data obtained in the study was performed using the SPSS 28.0 program. Mean, standard deviation, median, lowest, highest, frequency, and ratio values were used in the descriptive statistics of the data. The distribution of variables was measured with the Kolmogorov–Smirnov and Shapiro–Wilk tests. The independent sample *t*-test was used in the analysis of quantitative independent data with normal distribution. The Mann–Whitney U test was used in the analysis of quantitative independent data with non-normal distribution. The chi-square test was used in the analysis of qualitative independent data, and Fischer’s exact test was used when chi-square test conditions were not met. Results were considered statistically significant if the *p*-value was ≤0.05.

## 3. Results

A total of 302 participants were included in the study, of whom 152 (50.3%) were male and 150 (49.7%) were female. Body Mass Index of participants (BMI)—Min–Max: 16.5–47.3; Median: 27.5; Mean ± Standard Deviation: 27.8 ± 5.0 (in Table 1). The demographic characteristics are displayed in Table 1 and the genetic and laboratory characteristics of all participants are presented in Table 2.

No significant differences (*p* > 0.05) were observed in the distribution of ApoC-III rs2854116/FokI and ApoC-III rs2854117/MspI genotypes between case and control groups. However, the case group had significantly higher (*p* < 0.05) FIB4 scores and MASLD fibrosis scores compared to the control group. Similarly, the risk stratification based on both scores showed significantly higher (*p* < 0.05) risk levels in the case group (Table 3).

When comparing patients with TT versus CT/CC genotypes of ApoC-III rs2854116/FokI, no significant differences (*p* > 0.05) were found in the presence of hepatic steatosis, liver cirrhosis, dyslipidemia, hypertriglyceridemia, hypercholesterolemia, diabetes status, or diabetes complications (nephropathy, retinopathy, neuropathy). Cardiovascular disease rates were also similar between these genotype groups (Table 4).

Laboratory parameters including CRP, AST, ALT, ALP, GGT, fasting glucose, HbA1c, HOMA-IR, insulin, C-peptide, LDL, triglycerides, HDL, total cholesterol, and AST/ALT ratio showed no significant differences between the genotype groups. However, LDH levels were significantly higher (*p* < 0.05) in the CT/CC group compared to the TT group. The CC genotype of ApoC-III rs2854117/MspI was significantly more common (*p* < 0.05) in the CT/CC group of ApoC-III rs2854116/FokI compared to the TT group. No significant differences were observed in FIB4 score, MASLD fibrosis score, or disease duration between these genotype groups.

Similarly, when comparing patients with CC versus CT/TT genotypes of ApoC-III rs2854117/MspI, no significant differences (*p* > 0.05) were found in the presence of hepatic steatosis, liver cirrhosis, dyslipidemia, hypertriglyceridemia, hypercholesterolemia, diabetes status, complications, or cardiovascular disease (Table 5 and Table 6).

All laboratory parameters and fibrosis scores were comparable between the ApoC-III rs2854117/MspI genotype groups, with no significant differences in disease duration.

## 4. Discussion

The major findings of this study indicate that the ApoC-III gene polymorphisms rs2854116 and rs2854117 are not significantly associated with the presence or severity of metabolic dysfunction-associated steatotic liver disease (MASLD) in our Turkish population. This contrasts with the expected metabolic alterations observed in MASLD patients, such as higher BMI, elevated liver enzymes (ALT, AST, GGT), increased triglyceride levels, and reduced HDL cholesterol, which were not linked to the ApoC-III genotypes in our cohort. Furthermore, neither polymorphism showed a significant correlation with steatosis grades observed on ultrasonography nor with non-invasive fibrosis scores, including FIB-4 and the MASLD fibrosis score.

MASLD has been associated with subclinical levels of systemic inflammation [26,27]. A meta-analysis demonstrated that patients with MASLD exhibit significantly elevated levels of C-reactive protein (CRP), along with increases in other inflammatory markers, thereby supporting the presence of subclinical inflammation in this patient population [28]. In our study, the observed elevation in CRP levels among individuals with MASLD further reinforces the notion that subclinical inflammation is a characteristic feature accompanying the disease.

When comparing the patient and control groups, the median fasting glucose level was found to be 95.5 mg/dL in the patient group and 86 mg/dL in the control group. Similarly, the median HbA1c level was 5.75% in the patient group and 5.20% in the control group, with the differences being statistically significant (*p* < 0.05). This disparity is likely attributable to the presence of individuals with comorbid conditions such as diabetes mellitus, impaired glucose tolerance, and impaired fasting glucose in the patient group, whereas the control group consisted of healthy adults. Furthermore, the higher levels of insulin and C-peptide observed in the patient group may be explained by the presence of hyperglycemia and type 2 diabetes in this group, as well as a higher prevalence of obesity and central obesity, which are commonly associated with increased insulin resistance.

In the evaluation of laboratory lipid profile parameters, triglyceride, total cholesterol, and LDL levels were found to be significantly higher in the patient group compared to the control group (*p* < 0.05). Plasma lipid abnormalities are more frequently observed in individuals with MASLD, and triglycerides (TG) play a critical role in the pathogenesis of the disease [29]. The differences in total cholesterol, LDL, and TG levels observed in our study are consistent with the existing literature and may reflect the metabolic burden and increased risk of metabolic syndrome in patients with MASLD.

A statistically significant difference was observed between the patient and control groups in liver enzyme levels, including AST, ALT, ALP, and GGT. Additionally, the AST/ALT ratio was significantly lower in the patient group. The median HOMA-IR value was 3.60 in the patient group compared to 1.65 in the control group. Considering that a HOMA-IR value of 2 is widely accepted as the threshold for insulin resistance, these findings indicate a higher prevalence of insulin resistance among individuals with MASLD [30]. This aligns with existing literature, which underscores the close association between MASLD and insulin resistance [4].

Among the non-invasive tests (NITs) used for assessing liver fibrosis, FIB-4 and NAFLD fibrosis score (NFS) levels were found to be higher in the MASLD group. When the FIB-4 and NFS scores were stratified into fibrosis risk categories, the relatively low number of patients in the high-risk group suggests that the patient population in this study represents individuals with a lower fibrosis burden. This implies that our study predominantly reflects patients in the earlier stages of MASLD. The observed correlation with FIB-4 and NFS provides insight into the relationship between liver fibrosis and more advanced stages of MASLD, including non-alcoholic steatohepatitis (NASH).

Polymorphisms in ApoC-III have shown inconsistent associations with MASLD across different populations in the literature. While meta-analyses suggest mixed results, some studies have reported that the rs2854116 polymorphism (T-455C) in the ApoC-III gene promoter may be associated with increased risk of MASLD, hypertriglyceridemia, and insulin resistance, particularly in Asian populations including South Asians, though the rs2854117 polymorphism (C-482T) shows less consistent associations. However, the clinical significance of these variants remains unclear due to conflicting findings across studies and populations [8,31].

Another study examining ApoC-III polymorphism in Asian countries, which included 1750 MASLD patients and 2181 healthy participants, reported a strong association between the rs2854116 variant and MASLD. However, in the subgroup analysis of MASLD cases by country, no relationship was detected in China [31]. In another study conducted in China with 390 MASLD patients and 409 healthy individuals, no significant difference was found between the MASLD population and controls in terms of genotype and allele frequencies of rs2854116 and rs2854117 (*p* > 0.05) [32]. In our study, the polymorphisms in ApoC-III (rs2854116 and rs2854117) examined in participants were not found to be significant between the patient group and the control group. This may be attributed to all participants being of Turkish ethnic origin.

Regarding the elevated lactate dehydrogenase (LDH) levels in rs2854116 CT/CC carriers in our study, LDH is a non-specific marker of tissue damage. While the precise pathophysiological relevance of this elevation in the context of MASLD for these specific genotypes requires further investigation, it may suggest increased cellular stress or damage that is not directly mediated by the ApoC-III variants’ primary role in lipid metabolism, or it could be a consequence of broader metabolic perturbations linked to these genotypes, even if not directly leading to MASLD susceptibility in our cohort.

### Limitations

Our study’s reliance on ultrasonography for MASLD diagnosis is a limitation. While widely used as a first-line method due to its non-invasive nature, ultrasonography is relatively insensitive for detecting mild steatosis, and liver biopsy or MRI-PDFF are considered more accurate modalities. This could affect the precision of steatosis grading and potentially influence associations. Additionally, while we analyzed the main ApoC-III variants, we did not investigate other SNPs such as PNPLA3, TM6SF2, GCKR, MBOAT7, or HSD17B13, which are extensively recognized for their significant contributions to MASLD susceptibility and progression. This limits the comprehensive understanding of genetic contributions in our cohort. The single-center design and limited representation of patients with advanced fibrosis and compensated cirrhosis, along with the absence of a positive control group for comorbid conditions, also limit the generalizability of our findings. Future studies with larger, multi-ethnic cohorts, utilizing gold-standard diagnostic methods, and incorporating a broader panel of genetic markers are warranted to fully elucidate the complex genetic architecture of MASLD in the Turkish population and globally.

## 5. Conclusions

This study represents the first comprehensive investigation of ApoC-III gene polymorphisms in Turkish MASLD patients, contributing valuable population-specific genetic data to the international literature. Our analysis of rs2854116 (T-455C) and rs2854117 (C-482T) polymorphisms revealed no significant associations between these variants and MASLD susceptibility in our Turkish population, contrasting with positive associations reported in other ethnic populations. Understanding these genetic contributions is crucial for developing personalized diagnostic tools and management strategies for MASLD, emphasizing the need for comprehensive genetic studies in diverse populations.

## Figures and Tables

**Table 1:** Demographic characteristics.

Participants Characteristics	Min–Max	Median	Mean ± SD	n (%)
**Age**	19.0–71.0	44.0	43.8 ± 15.0	
**Basal Metabolic Age**	17.0–79.0	47.0	46.3 ± 14.5	
**Gender**				
* Female*				150 (49.7%)
* Male*				152 (50.3%)
**Height (m)**	1.46–1.94	1.69	1.70 ± 0.10	
**Weight (kg)**	46.0–130.0	81.0	80.4 ± 15.9	
**Body Mass Index(BMI) (kg/m^2^)**	16.5–47.3	27.5	27.8 ± 5.0	
* Underweight*				5 (1.7%)
* Normal*				86 (28.5%)
* Overweight*				121 (40.1%)
* Obese*				90 (29.8%)
**Body Density**	0.96–1.07	1.02	1.02 ± 0.02	
**Fat Percentage %**	10.0–56.0	29.0	30.1 ± 8.6	
**Water Percentage %**	1.0–69.0	51.0	50.7 ± 7.8	
**Waist Circumference (cm)**	59.0–136.0	98.0	99.5 ± 13.6	
**Neck Circumference (cm)**	24.0–98.0	36.0	36.2 ± 6.1	
**Waist/Height Ratio**	0.36–0.80	0.55	0.56 ± 0.08	
**Leg Circumference (cm)**	28.0–70.0	48.0	47.5 ± 6.8	
**Arm Circumference (cm)**	17.0–91.0	28.0	28.3 ± 5.0	
**Active Smoking**				
* No*				210 (69.5%)
* Yes*				92 (30.5%)
**Cigarette Consumption (Pack/Year)**	1.0–90.0	15.0	19.7 ± 19.2	
**Ex-Smoker**				
* No*				281 (93.0%)
* Yes*				21 (7.0%)
**Ex-Smoker Duration (Months)**	1.0–360.0	120.0	136.8 ± 112.9	
**Alcohol Use**				
* No*				245 (81.1%)
* Yes*				57 (18.9%)
**Alcohol Consumption (Day/Week)**	0.0–175.0	25.0	53.8 ± 51.6	
**Systolic Blood Pressure (mmHg)**	94.0–158.0	126.0	125.7 ± 11.6	
**Diastolic Blood Pressure (mmHg)**	39.0–101.0	79.0	79.2 ± 9.4	

**Table 2:** Demographic characteristics and ApoC-III alleles of all participants.

Variable	Category	n	%
USG Steatosis	(−)	102	33.8%
	Grade I	91	30.1%
	Grade II	93	30.8%
	Grade III	16	5.3%
Liver Cirrhosis	(−)	301	99.7%
	(+)	1	0.3%
Dyslipidemia	(−)	166	55.0%
	(+)	136	45.0%
Hypertriglyceridemia	(−)	230	76.2%
	(+)	72	23.8%
Hypercholesterolemia	(−)	202	66.9%
	(+)	100	33.1%
Diabetes Status	(−)	209	69.2%
	Diabetes	62	20.5%
	Impaired Fasting Glucose	26	8.6%
	Impaired Glucose Tolerance	5	1.7%
Nephropathy	(−)	295	97.7%
	(+)	7	2.3%
Retinopathy	(−)	297	98.3%
	(+)	5	1.7%
Neuropathy	(−)	294	97.4%
	(+)	8	2.6%
Cardiovascular Disease	(−)	293	97.0%
	(+)	9	3.0%
ApoC-III rs2854116/Fokl	CC	94	31.1%
	TC	143	47.4%
	TT	65	21.5%
ApoC-III rs2854117 Mspl	CC	135	44.7%
	TC	108	35.8%
	TT	59	19.5%
**FIB4 Score**	0.21–42.16	0.70	1.00 ± 0.70
**FIB4 Risk Categories**			
Low Risk			269 (89.1%)
Intermediate Risk			29 (9.6%)
High Risk			4 (1.3%)
**MASLD Fibrosis Score**	−6.07–1.45	−2.61	−2.58 ± 1.42
**MASLD Risk Categories**			
Low Risk			241 (79.8%)
Intermediate Risk			57 (18.9%)
High Risk			4 (1.3%)

**Note:** Diagnostic criteria for diabetes mellitus include a Hemoglobin A1C of 6.5% or higher, a fasting glucose at or above 126 mg/dL, or a random glucose of 200 mg/dL or greater with symptoms. Dyslipidemia is defined by blood lipid levels such as an LDL cholesterol of 160 mg/dL or higher, an HDL cholesterol below 40 mg/dL, or triglycerides at or above 200 mg/dL. Nephropathy, or chronic kidney disease, is established when a patient’s GFR is below 60 or albuminuria (ACR ≥ 30 mg/g) persists for more than three months. Diabetic retinopathy is staged based on fundoscopic findings, progressing from non-proliferative disease with microaneurysms to proliferative disease marked by neovascularization. The criteria for liver cirrhosis involve a combination of clinical signs, specific laboratory abnormalities, and imaging findings, with a biopsy providing definitive confirmation. For cardiovascular disease, Stage 2 hypertension is a key criterion, defined by a blood pressure reading of 140/90 mm Hg or higher. −: ABSENT, +: EXIST.

**Table 3:** Comparison of genetic profiles and scores between control and case groups.

Variable	Category	Control Group (n = 100)				Case Group (n = 202)				*p*-Value	Test
		n	%	Mean ± SD	Median	n	%	Mean ± SD	Median		
ApoC-III rs2854116/FokI	CC	31	31.0%	-	-	63	31.2%	-	-	0.990	X^2^
	TC	47	47.0%	-	-	96	47.5%	-	-		
	TT	22	22.0%	-	-	43	21.3%	-	-		
ApoC-III rs2854117 MspI	CC	46	46.0%	-	-	89	44.1%	-	-	0.543	X^2^
	TC	38	38.0%	-	-	70	34.7%	-	-		
	TT	16	16.0%	-	-	43	21.3%	-	-		
FIB4 Score	-	-	-	0.84 ± 2.18	0.57	-	-	1.09 ± 2.94	0.80	0.000	m
FIB4 Score Categories	Low Risk	97	97.0%	-	-	172	85.1%	-	-	0.002	X^2^
	Moderate Risk	2	2.0%	-	-	27	13.4%	-	-		
	High Risk	1	1.0%	-	-	3	1.5%	-	-		
MASLD Fibrosis Score	-	-	-	−3.47 ± 1.02	−3.67	-	-	−2.14 ± 1.39	−2.16	0.000	m
MASLD Fibrosis Score Categories	Low Risk	97	97.0%	-	-	144	71.3%	-	-	0.000	X^2^
	Moderate Risk	3	3.0%	-	-	54	26.7%	-	-		
	High Risk	0	0.0%	-	-	4	2.0%	-	-		

**Abbreviations:** mean, average; SD—standard deviation; t—independent sample *t*-test; m—Mann–Whitney U test; X^2^—chi-square test (Fisher’s test).

**Table 4:** Comparison of clinical and laboratory characteristics between patients with TT and CT/CC genotypes of the ApoC-IIIrs2854116/FokI variant.

Parameter	Category	TT (n = 43)		CT/CC (n = 159)		*p*-Value/Test
		n	%	n	%	
**USG Steatosis**	(−)	0	0.0%	3	1.9%	1.000/X^2^
	Grade I	18	41.9%	72	45.3%	
	Grade II	21	48.8%	72	45.3%	
	Grade III	4	9.3%	12	7.5%	
**Liver Cirrhosis**	(−)	43	100.0%	158	99.4%	1.000/X^2^
	(+)	0	0.0%	1	0.6%	
**Dyslipidemia**	(−)	16	37.2%	78	49.1%	0.167/X^2^
	(+)	27	62.8%	81	50.9%	
**Hypertriglyceridemia**	(−)	26	60.5%	112	70.4%	0.212/X^2^
	(+)	17	39.5%	47	29.6%	
**Hypercholesterolemia**	(−)	23	53.5%	97	61.0%	0.373/X^2^
	(+)	20	46.5%	62	39.0%	
**Diabetes Status**	(−)	24	55.8%	86	54.1%	0.840/X^2^

−: ABSENT, +: EXIST.

**Table 5:** Comparison of clinical and laboratory characteristics between patients with CC and CT/TT genotypes of ApoC-IIIrs2854117/MspI variant.

Category	Group	CC (n = 89)		CT/TT (n = 113)		*p*-Value/Test
		n	%	n	%	
Ultrasound Steatosis	No Steatosis	2	2.2%	1	0.9%	0.584 X^2^
	Grade I	41	46.1%	49	43.4%	
	Grade II	37	41.6%	56	49.6%	
	Grade III	9	10.1%	7	6.2%	
Liver Cirrhosis	No	89	100.0%	112	99.1%	1.000 X^2^
	Yes	0	0.0%	1	0.9%	
Dyslipidemia	No	43	48.3%	51	45.1%	0.653 X^2^
	Yes	46	51.7%	62	54.9%	
Hypertriglyceridemia	No	59	66.3%	79	69.9%	0.583 X^2^
	Yes	30	33.7%	34	30.1%	
Hypercholesterolemia	No	51	57.3%	69	61.1%	0.589 X^2^
	Yes	38	42.7%	44	38.9%	
Diabetes Status	No	45	50.6%	65	57.5%	0.436 X^2^
	Diabetes	30	33.7%	32	28.3%	
	Impaired Fasting	13	14.6%	12	10.6%	
	Impaired Glucos	1	1.1%	4	3.5%	
Nephropathy	No	86	96.6%	109	96.5%	0.948 X^2^
	Yes	3	3.4%	4	3.5%	
Retinopathy	No	85	95.5%	112	99.1%	0.101 X^2^
	Yes	4	4.5%	1	0.9%	
Neuropathy	No	85	95.5%	109	96.5%	0.730 X^2^
	Yes	4	4.5%	4	3.5%	
Cardiovascular Disease	No	84	94.4%	109	96.5%	0.477 X^2^
	Yes	5	5.6%	4	3.5%	

**Table 6:** Comparison of laboratory characteristics between patients with CC and CT/TT genotypes of ApoC-IIIrs2854117/MspI variant.

Parameter	CC (Mean ± SD)	CC (Median)	CT/TT (Mean ± SD)	CT/TT (Median)	*p*-Value
CRP	3.15 ± 3.26	2.10	3.39 ± 4.26	2.40	694
AST	23.0 ± 8.8	21.5	26.1 ± 19.3	22.0	665
ALT	32.2 ± 22.5	25.9	34.1 ± 29.5	26.9	842
ALP	77.1 ± 25.0	75.0	76.9 ± 21.8	76.0	688
GGT	30.1 ± 22.0	23.0	32.7 ± 29.1	26.0	558
LDH	191.8 ± 41.4	183.0	190.9 ± 47.6	180.0	665
Fasting Glucose	107.4 ± 33.8	95.0	106.9 ± 40.4	96.0	575
HbA1c	6.14 ± 1.39	5.80	6.08 ± 1.38	5.70	623
Homa-IR	5.33 ± 8.12	3.46	4.71 ± 5.11	3.67	561
Insulin	18.4 ± 17.3	14.5	16.9 ± 13.8	15.0	881
C-Peptide	3.77 ± 2.30	3.43	3.55 ± 1.68	3.40	722
LDL	123.4 ± 33.1	122.0	127.6 ± 40.4	126.0	557
Triglyceride	177.9 ± 119.8	138.2	168.2 ± 113.5	142.0	421
HDL	47.8 ± 16.4	44.4	45.1 ± 11.8	44.0	393
Total Cholesterol	195.3 ± 36.7	190.0	198.7 ± 49.2	192.0	686
AST/ALT Ratio	0.86 ± 0.31	0.85	0.91 ± 0.37	0.85	553
Disease Duration (Month)	33.1 ± 68.6	0.0	23.5 ± 50.9	0.0	564

## Data Availability

The original contributions presented in this study are included in the article. Further inquiries can be directed to the corresponding author.
